# Autonomous Navigation of Mobile Robots: A Hierarchical Planning–Control Framework with Integrated DWA and MPC

**DOI:** 10.3390/s25072014

**Published:** 2025-03-23

**Authors:** Zhongrui Wang, Shuting Wang, Yuanlong Xie, Tifan Xiong, Chao Wang

**Affiliations:** 1 School of Mechanical Science and Engineering, Huazhong University of Science and Technology, Wuhan 430074, China; wangzr@hust.edu.cn (Z.W.); wangst@hust.edu.cn (S.W.); yuanlongxie@hust.edu.cn (Y.X.); xiongtf@hust.edu.cn (T.X.); 2 State Key Laboratory of Robotics and Systems, Harbin Institute of Technology, Harbin 150080, China

**Keywords:** mobile robot, path planning, tracking control, Dynamic Window Approach, Model Predictive Control

## Abstract

In human–robot collaborative environments, the inherent complexity of shared operational spaces imposes dual requirements on process safety and task execution efficiency. To address the limitations of conventional approaches that decouple planning and control modules, we propose a hierarchical planning–control framework. The proposed framework explicitly incorporates path tracking constraints during path generation while simultaneously considering path characteristics in the control process. The framework comprises two principal components: (1) an enhanced Dynamic Window Approach (DWA) for the local path planning module, introducing adaptive sub-goal selection method and improved path evaluation functions; and (2) a modified Model Predictive Control (MPC) for the path tracking module, with a curvature-based reference state online changing strategy. Comprehensive simulation and real-world experiments demonstrate the framework’s operational advantages over conventional methods.

## 1. Introduction

The maturation of robotics and cyber-physical systems has propelled mobile robots into critical roles across smart manufacturing and logistics ecosystems [1,2,3,4]. These autonomous platforms now constitute essential infrastructure for material handling and process automation in industrial production and warehouse management [5]. These operational environments typically exhibit dynamic uncertainty characterized by static and dynamic obstacles, including misplaced components or moving humans [6,7]. These dynamic interactions within the environment frequently interfere with the intended global trajectories of mobile robots, rendering the predefined paths ineffective [8,9]. Conventional fixed-path tracking systems prove inadequate in such non-stationary conditions, severely compromising operational efficiency and limiting industrial applications. Thus, to maintain operational continuity during different tasks, mobile robots must simultaneously achieve closed-loop trajectory following through advanced tracking controllers and real-time local reference path generation upon obstacle detection. This integration of real-time path re-planning with robust trajectory tracking forms the foundational requirement for the safe autonomous navigation of mobile robots.

The Dynamic Window Approach (DWA), a widely implemented path planning algorithm, determines an optimal local path through real-time fusion of sensor-acquired environmental data and robotic odometric information [10,11]. This methodology operates via four computational phases: (1) establishing a velocity window by enumerating all kinematically feasible velocities within current dynamic constraints; (2) discretizing the velocity domain through systematic sampling; (3) generating trajectory predictions; and (4) executing multi-objective optimization through predefined objective functions to identify the optimal trajectory for subsequent actuation [12,13]. Ref. [14] introduces the Finite Distribution Estimation-based DWA (FDEDWA), which enhances DWA by integrating finite memory filtering (FMF) to estimate obstacle distributions in dynamic environments, enabling robust navigation through noise reduction via Frobenius norm optimization and covariance adaptation. A hybrid navigation method that combines reinforcement learning with the DWA is proposed in [15], employing a neural network trained via proximal policy optimization (PPO) to dynamically adjust DWA’s cost function weights based on short-term obstacle motion observations. Ref. [16] presents a hybrid algorithm for Automated Guided Vehicles (AGVs) in Industry 4.0 settings, combining an enhanced Social Force Model (SFM) and improved Dynamic Window Approach (DWA) with SparkLink communication technology to optimize dynamic obstacle avoidance by boosting environmental perception and data reliability. Ref. [17] proposes a dual-layer path planning algorithm for multi-robot systems in dynamic environments, integrating an improved Glasius Bio-inspired Neural Network (GBNN) with an enhanced DWA to optimize global path planning and enable real-time obstacle avoidance. However, existing DWA planning methods exhibit critical limitations in concurrently ensuring trajectory smoothness and planning reliability. A fundamental limitation stems from inadequately addressing the geometric continuity between local and global path segments and neglecting the kinematic constraint of the system. This dual deficiency induces geometrically non-smooth trajectories, thereby triggering unstable maneuvers during obstacle avoidance.

Trajectory tracking control constitutes a closed-loop process where controllers compute actuation commands (e.g., velocity and torque) based on the state error between the robotic system’s current state and the reference trajectory, subsequently driving wheel motors to achieve path-following precision [18,19]. Distinguished by its model-based optimization framework, Model Predictive Control (MPC) operates through three constitutive elements: predictive model, receding horizon optimization, and feedback compensation mechanisms [20,21]. This approach demonstrates superior robustness and control accuracy compared to conventional methods, especially in handling constrained optimization through real-time quadratic programming that explicitly incorporates system constraints (e.g., torque saturation and velocity boundaries) [22,23]. This constraint-handling capability, absent in most tracking controllers, renders MPC increasingly popular in real-world applications [24,25]. Ref. [26] combines MPC and Sliding Mode Control (SMC) to enhance path tracking precision and robustness by integrating sliding mode functions into the MPC framework and adaptively adjusting prediction/control horizons via fuzzy rules based on vehicle speed and path curvature. A nonlinear MPC framework for unmanned sailboats is proposed in [27], which jointly regulates velocity and heading, employing a Line-of-Sight (LOS) guidance with Lyapunov-based backstepping for non-upwind tracking and a zigzag strategy with lateral error-triggered heading switching for upwind scenarios. Ref. [28] introduces a parallel NMPC path tracking controller for autonomous vehicles, combining a Newton optimization algorithm with FPGA hardware acceleration to enhance real-time performance by simplifying the optimization problem through variable approximations and optimizing hardware design for speed–resource efficiency. A tube-based robust MPC for autonomous articulated vehicles is proposed in [29], combining a simplified lumped dynamics model with constraint integration to enhance path tracking accuracy and stability while ensuring robustness against modeling errors and worst-case disturbances. However, the inherent geometric particularity of local paths relative to global paths in autonomous navigation systems presents significant challenges for precise tracking control. Prevailing methodologies simplify controller architecture by employing identical control policies across both the nominal tracking mode and the obstacle avoidance mode. These methodologies prove inadequate when local path planning necessitates abrupt curvature variations (e.g., sharp turns) to circumvent obstacles, violating fundamental requirements for motion smoothness and tracking accuracy. To ensure system stability under such non-uniform curvature conditions, advanced control strategies must adaptively modulate control gains based on real-time path geometry characteristics such as the curvature.

**Figure 1:**
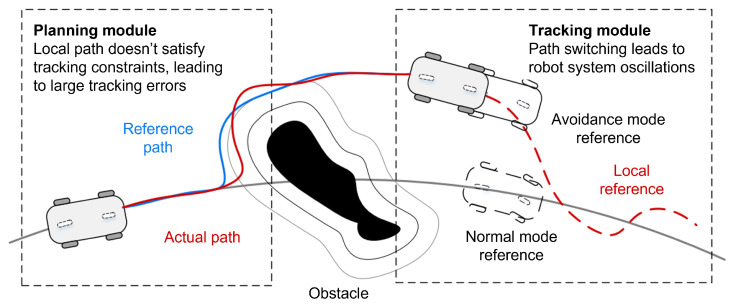
Shortcomings of current hierarchical frameworks.

Based on the above discussion, existing hierarchical frameworks typically neglect information integration between the local path planning and tracking stages. Thus, as shown in Figure 1, these methods have two critical limitations: (1) the control layer, uninformed by planning constraints, fails to adapt to trajectory modifications during tracking, potentially inducing system oscillations during mode transitions (e.g., normal operation to obstacle avoidance); and (2) the planned trajectories, generated without considering control dynamics, may violate the robot’s kinematic and dynamic characteristics, resulting in significant tracking errors that could precipitate unintended collisions. These limitations fundamentally lead to poor autonomous navigation performance of mobile robotic systems in dynamic environments. Therefore, navigation with simultaneous consideration of control and planning is of research value. Consequently, we proposed a framework for robotic navigation that integrates the control and planning stages in this paper. The following issues are considered:

1. For better local planning performance, the selection of a local target requires careful consideration of the spatial relationships of the robot and the obstacle to ensure effective navigation. Additionally, enhancing path smoothness through geometric continuity constraints is essential for maintaining control stability during obstacle avoidance maneuvers. In addition, guaranteeing planning success rates in complex environments necessitates adaptive methods that preserve path connectivity between the start and goal points under varying obstacle situations.
2. How to design an effective controller that will ensure smooth transitions between global and local paths for the robot to achieve high tracking accuracy and operational safety.

**Figure 2:**
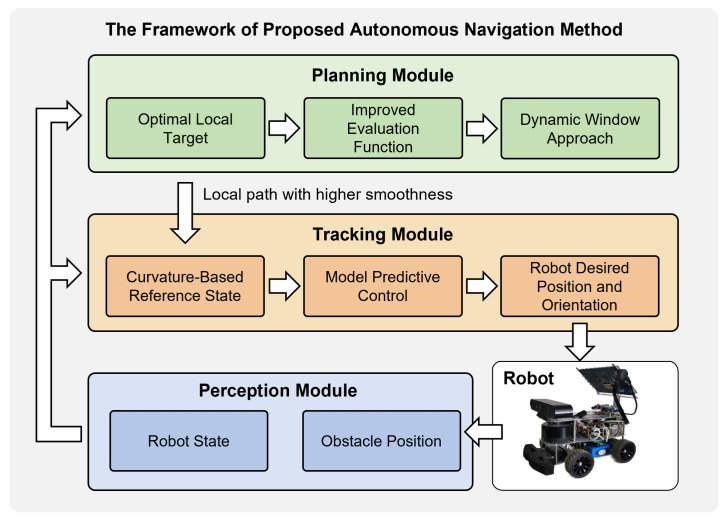
The framework of the proposed autonomous navigation method.

The key contribution of this paper are as follows:

1. A hierarchical planning–control framework with integrated DWA and MPC was proposed. The proposed framework holistically considers trajectory feasibility, continuity during path recovery, and tracking accuracy. The overall scheme is shown in Figure 2.
2. For the local path planning module, an online local target point selection strategy was developed to address the limitations of conventional planning methods that inadequately consider the correlation between local and global paths, while simultaneously improving planning success rates across diverse environments. Additionally, building upon the standard DWA, we improved the path smoothness through modifications to the path evaluation function. These enhancements provide convenience for the subsequent control module.
3. Regarding the trajectory tracking control module, a curvature-adaptive reference point adjustment mechanism was proposed to mitigate tracking accuracy degradation in the obstacle avoidance mode. Furthermore, we implemented adaptive optimization of both prediction horizon and control horizon parameters, thereby significantly improving the tracking precision and the computational efficiency of the controller.

The rest of this paper is organized as follows. Section 2 gives the preliminaries of the proposed method. Then, the hierarchical planning-control framework with integrated DWA and MPC is introduced in detail in Section 3. In Section 4, a series of simulations and real-world experiments is conducted to demonstrate the advantages of the proposed framework over traditional methods. Finally, Section 5 concludes this paper.

## 2. Preliminaries

### 2.1. The Dynamic Window Approach

**Figure 3:**
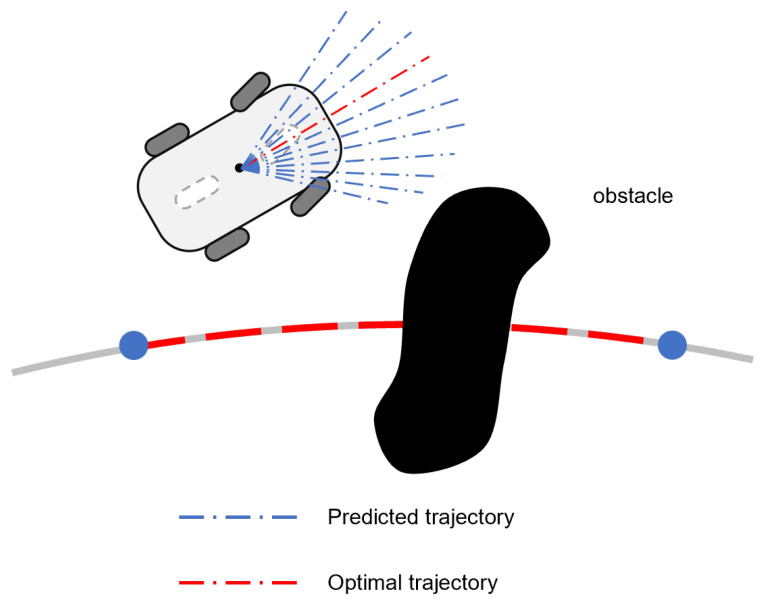
Main steps of DWA: velocity sampling, trajectory prediction, and trajectory evaluation.

DWA is a well-established local path planning methodology capable of generating collision-free trajectories, demonstrating particular effectiveness in real-time navigation scenarios [30,31]. The approach operates within a velocity window defined by angular velocity ω and linear velocity *v* parameters. The evaluation function simultaneously optimizes for obstacle avoidance, motion smoothness, and target convergence. The velocity vector achieving maximum evaluation score is selected as the optimal velocity command for subsequent planning stages. Through this iterative process, DWA progressively constructs collision-free trajectories from initial position to the designated target [32], as shown in Figure 3. The subsequent control layer then executes trajectory tracking commands derived from this planned path, ultimately achieving collision-free navigation through coordinated planning–control interactions.

**Figure 4:**
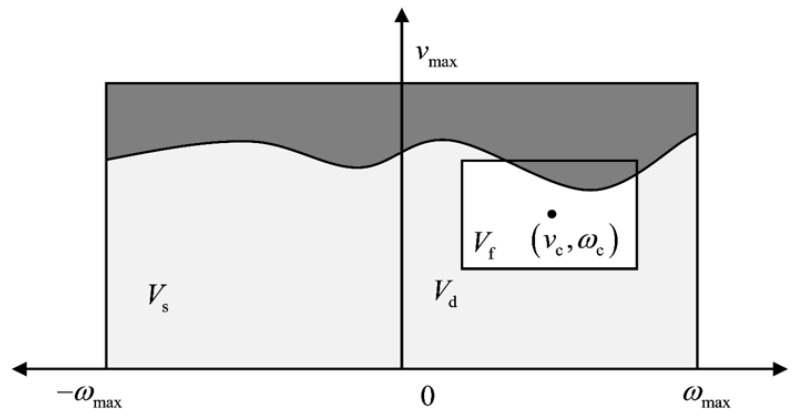
The velocity window.

Velocity sampling fundamentally involves the systematic construction of an admissible velocity window through a multi-constraint synthesis to satisfy application-specific operational requirements. As illustrated in Figure 4, the velocity window $V_{f}$ of mobile robots during a certain sampling period can be expressed as:

(1) $$V_{f}=V_{s}⋂V_{d}⋂V_{a}$$

The primary constraints originate from bounded ranges defined by the linear velocity and angular velocity limits, which constitute the fundamental boundaries of the velocity window $V_{s}$:

(2) $$V_{s}=\left\{\left(v,\omega \right)\left|\begin{array}{c} v\in \left[0,v_{\max}\right]\\ \omega \in \left[-\omega_{\max},\omega_{\max}\right] \end{array}\right.\right\}$$

Given a current velocity state $\left(v_{c},\omega_{c}\right)$, the dynamically admissible velocity window $V_{d}$ arises from bounded acceleration capabilities, specifically the maximum linear acceleration ${\dot{v}}_{m}$ and angular acceleration ${\dot{\omega }}_{m}$, constrained by the torque limitations of the drive system:

(3) $$V_{d}=\left\{\left(v,\omega \right)\left|\begin{array}{c} v\in \left[v_{c}-{\dot{v}}_{m}\Delta t,v_{c}+{\dot{v}}_{m}\Delta t\right]\\ \omega \in \left[\omega_{c}-{\dot{\omega }}_{m}\Delta t,\omega_{c}+{\dot{\omega }}_{m}\Delta t\right] \end{array}\;\;\;\;\right.\right\}$$

The final constraint stems from collision-avoidance requirements, mandating the capability to execute an emergency stop before potential collisions. This safety criterion defines the admissible velocity window $V_{a}$ through the braking condition:

(4) $$V_{a}=\left\{\left(v,\omega \right)\left|\begin{array}{c} v\in \left[0,\sqrt{2\cdot \mathrm{dist}\left(v,\omega \right)\cdot {\dot{v}}_{m}}\right]\\ \omega \in \left[0,\sqrt{2\cdot \mathrm{dist}\left(v,\omega \right)\cdot {\dot{\omega }}_{m}}\right] \end{array}\right.\right\}$$

The computed velocity window $V_{f}$ undergoes systematic discretization to generate candidate velocity pairs. These discretized (v,ω) values are subsequently applied as control inputs to predict states through the system model, yielding all kinematically feasible trajectories at the current planning iteration. The trajectory evaluation process employs a multi-criteria framework where all candidate trajectories undergo quantitative assessment. Finally the velocity (v,ω) corresponding to the trajectory with the highest score is selected as the simulated velocity at the next planning moment. The fundamental optimization objective combines obstacle avoidance with time-optimal navigation, mathematically expressed as minimizing the time-to-goal $t_{f}$ under safety constraints:

(5) $$f\left(v,\omega \right)=\rho \left(w_{h}\cdot \mathrm{heading}\left(v,\omega \right)+w_{v}\cdot \mathrm{vel}\left(v,\omega \right)+w_{o}\cdot \mathrm{obdist}\left(v,\omega \right)\right)$$

where $w_{h}$, $w_{v}$ and $w_{o}$ are the weighting coefficients of each sub-evaluation function, and $\rho \left(\cdot \right)$ denotes the normalization function. The specific form of each sub-evaluation function can be expressed as follows:

(6) $$\mathrm{heading}\left(v,\omega \right)=180{}^{\circ}-\Delta \theta$$

(7) $$\mathrm{vel}\left(v,\omega \right)=\left|vs.\right|$$

(8) $$\mathrm{obdist}\left(v,\omega \right)=\left\{\begin{array}{ccc} d_{\max} & , & d_{\mathrm{obs}}\geq \tau_{\mathrm{obs}}\\ d_{\mathrm{obs}} & , & d_{\mathrm{obs}}<\tau_{\mathrm{obs}} \end{array}\right.$$

where Δθ and $d_{\mathrm{obs}}$ represents the target direction vector and distance value from the terminus of the trajectory to be assessed to the local navigation target, respectively. $d_{\max}$ and $\tau_{\mathrm{obs}}$ are predefined parameters with respect to the obstacle avoidance range and the maximum evaluation threshold.

### 2.2. System Modeling

**Figure 5:**
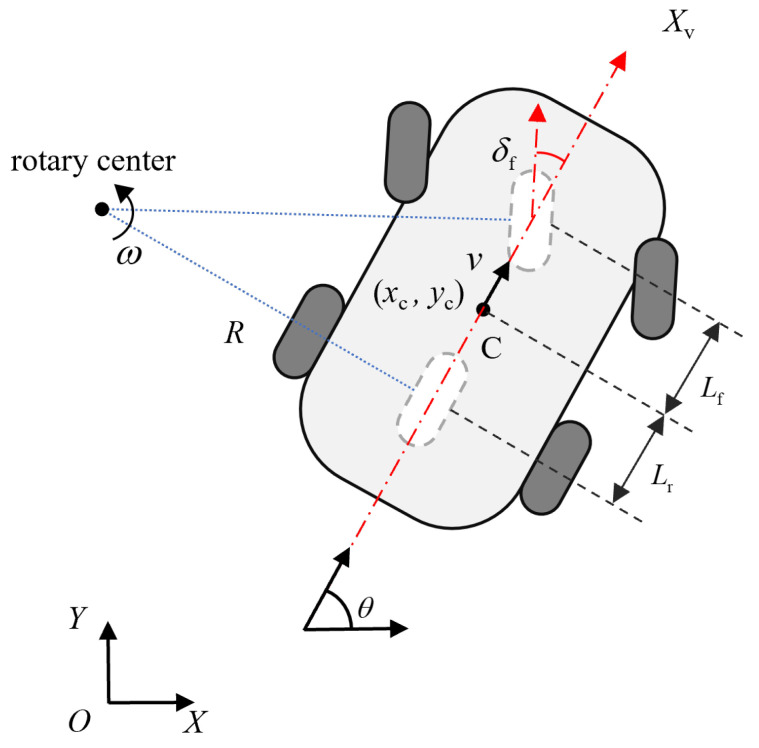
The kinematic model of the robot system.

Under low-velocity operational conditions, kinematic modeling demonstrates superior fidelity in characterizing a system while maintaining structural simplicity, thus achieving prevalent adoption in mobile robotic systems. The kinematic model investigates the geometric dependencies governing mobile robots’ spatial-temporal behavior, incorporating analytical descriptions of positional coordinates, velocity profiles, and their temporal variations. Therefore, this paper employs the kinematic model to systematically examine motion planning and control strategies for autonomous navigation. As shown in Figure 5, let *XOY* denote the world coordinate system. Designating *C* as the robot’s center of mass, $\left(x_{c},y_{c}\right)$ corresponds to its Cartesian coordinates in the *XOY* frame. The $X_{v}$-axis orientation aligns with the robot’s heading direction, with θ specifying the angle between the $X_{v}$-axis and the global *X*-axis, formally defined as the yaw angle. The steering angle is denoted by $\delta_{f}$, while *v* and ω represent the linear velocity and angular velocity, respectively. $L_{f}$ and $L_{r}$, respectively, indicate the longitudinal distances from the front/rear axles to the center of mass. The instantaneous center of rotation (ICR), determined by the intersection of virtual front-wheel steering axis and rear-wheel alignment perpendicular, generates the turning radius *R* measured from the vehicle’s central axis. Based on the above definition, the kinematic model of the mobile robot can be represented as:

(9) $$\dot{\eta }=\left[\begin{array}{c} {\dot{x}}_{c}\\ {\dot{y}}_{c}\\ \dot{\theta } \end{array}\right]=\left[\begin{array}{c} \begin{array}{c} v\cos\theta \\ v\sin\theta \end{array}\\ \frac{\tan\delta_{f}}{L_{f}+L_{r}}v \end{array}\right]=f\left(\eta ,u\right)$$

with $\omega =\dot{\theta }=\frac{v}{R}=\frac{v}{L_{f}+L_{r}}\tan\delta_{f}$. $\eta ={\left[x_{c},y_{c},\theta \right]}^{T}$ and $u={\left[v,\omega \right]}^{T}$ denotes the state vector and control input, respectively.

## 3. Methodology

To enable autonomous navigation of mobile robots, this section proposes a hierarchical framework integrating local path planning with trajectory tracking control. The framework architecture was designed with two principal enhancements: (1) optimization of the evaluation function and sub-target selection strategy in the standard DWA to ensure trajectory feasibility and control compatibility; and (2) development of a curvature-adaptive model predictive controller incorporating reference point adjustment mechanisms to address path tracking inaccuracies caused by global–local path discrepancies.

### 3.1. The Improved DWA

There are clear options for improving the traditional DWA. Firstly, the existence of the trajectory evaluation function gives the designer a high degree of freedom to modify and optimize according to the different tasks; secondly, as a point-to-point path planning algorithm, the selection of the local sub-target points has a great influence on the planning results. Therefore, this section will propose an improvement scheme for these two points, so that the planned local paths are more in line with the needs of practical trajectory tracking control.

#### 3.1.1. Modification of the Evaluation Function

**Figure 6:**
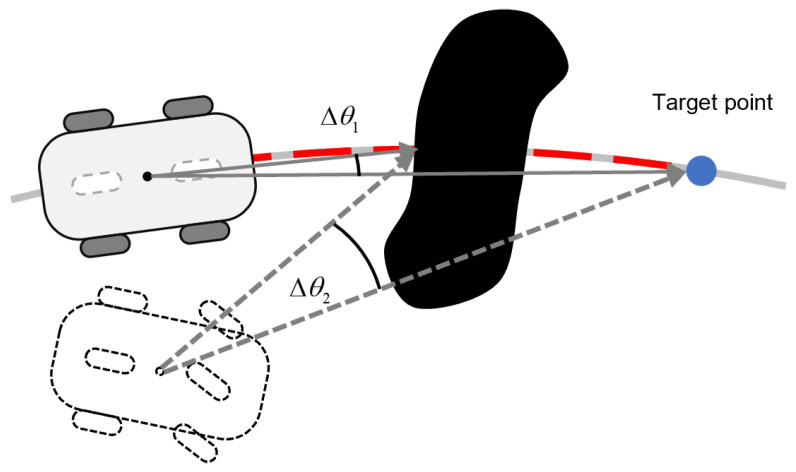
The conflict between heading evaluation and obstacle avoidance evaluation.

The conventional DWA exhibits inherent conflicts between its obstacle avoidance and heading alignment criteria. As illustrated in Figure 6, when obstacles proximate the target location, the heading evaluation prioritizes trajectories minimizing angular deviation Δθ, while the obstacle metric conversely favors trajectories maximizing clearance distance. This fundamental contradiction becomes particularly acute when obstacles obstruct the direct path to the goal, where the competing objectives induce oscillatory behavior that compromises system stability and may ultimately result in planning failures.

To mitigate path oscillations caused by the interference between heading angle and obstacle evaluation functions in mobile robot navigation, the conventional heading angle evaluation function is transformed into a target distance-based evaluation function godist(v,ω)

(10) $$\mathrm{godist}\left(v,\omega \right)=\frac{1}{\sqrt{{\left(x_{p}-x_{g}\right)}^{2}+{\left(y_{p}-y_{g}\right)}^{2}}+\varsigma }$$

where ς is a non-zero small quantity, and $\left(x_{p},y_{p}\right)$ and $\left(x_{g},y_{g}\right)$ represent the coordinates of the end of the predicted trajectory and the target point under the global coordinate system, respectively.

**Remark 1.**  *The target distance evaluation function is defined as the reciprocal of the distance between predicted trajectory and target coordinates. This metric demonstrates inverse proportionality characteristics: during long-range navigation phases, its minimal contribution to the overall evaluation function allows flexible path exploration, whereas near obstacles, the function dominates the optimization process to prioritize the dual objectives of target approach and obstacle avoidance. This dynamic mechanism effectively resolves the oscillation phenomenon inherent in conventional heading-based DWA.*

Secondly, the conventional method’s inherent limitation lies in its disregard for velocity continuity between consecutive planning cycles. The current optimal velocity vector (v,ω) is determined independently from the preceding state $\left(v_{\mathrm{prev}},\omega_{\mathrm{prev}}\right)$, creating discontinuous velocity variations. Through the curvature relationship k=ω/v, such discontinuities propagate into trajectory curvature fluctuations that exceed actuator tracking capabilities. To ensure smooth motion execution, the evaluation must incorporate curvature continuity constraints quantified by the curvature differential $\Delta k=|k_{t}-k_{t-1}|$, where lower differential values correspond to higher trajectory quality ratings:

(11) $$\mathrm{curcon}\left(v,\omega \right)=\frac{1}{\left|\frac{\omega }{v}-\frac{\omega_{\mathrm{last}}}{v_{\mathrm{last}}}\right|+\varsigma }$$

In summary, the improved evaluation function is:

(12) $$G\left(v,\omega \right)=\rho \left(w_{d}\cdot \mathrm{godist}\left(v,\omega \right)+w_{v}\cdot \mathrm{vel}\left(v,\omega \right)+w_{o}\cdot \mathrm{obdist}\left(v,\omega \right)+w_{c}\cdot \mathrm{curcon}\left(v,\omega \right)\right)$$

#### 3.1.2. Adaptive Sub-Target Selection Strategy

As a point-to-point navigation methodology, the determination of the sub-target critically influences planning performance. Optimal sub-target selection ensures trajectory continuity and smoothness, whereas bad choices may induce terminal oscillations or planning failures. Conventional fixed-interval sampling sub-targeting along global paths, while computationally efficient, demonstrates scenario sensitivity due to its static configuration. Particularly in cluttered environments where the obstacle distribution is stochastic, this approach suffers from robustness deficiencies and reduced success rates, exemplified by planning failure when obstacles proximate the sampled sub-target. To address these limitations, we propose an adaptive sub-target selection strategy. All candidate nodes within the target region undergo a multi-criteria cost analysis, with the optimal node selected through minimum-cost optimization. Notably, this methodology maintains algorithm-agnostic characteristics, enabling integration with various local planners beyond the baseline DWA.

The local sub-target determination process initiates with global path discretization. Given a predefined global trajectory ξ spanning from initial position $p_{s}$ to terminal goal $p_{g}$, we construct a discrete reference point set through parametric sampling:

(13) $$\xi =\left\{p_{s},p_{2},p_{3}\ldots p_{g}\right\}$$

where $p_{i}\in R^{2}$ denotes Cartesian coordinates.

**Figure 7:**
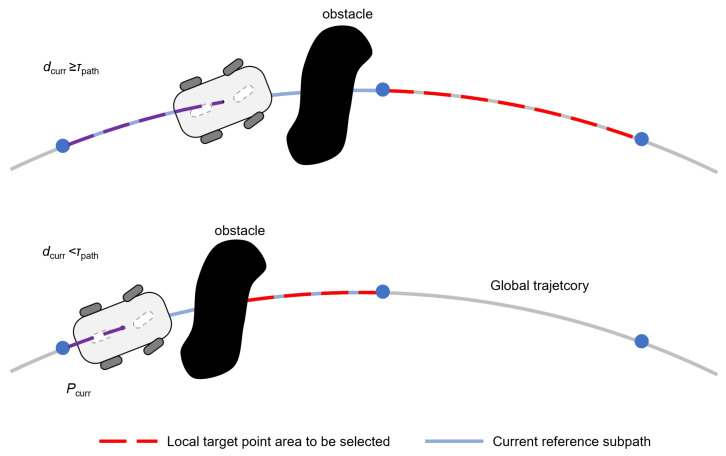
The viable target region with a threshold-based switching strategy.

Building upon the fixed-interval sampling method, the global path undergoes uniform segmentation into *m* isomorphic sub-paths $\xi_{j}\left(j=1,\ldots ,m\right)$, each maintaining equal arc length Δs. This decomposition transforms the holistic navigation task into sequential sub-goal tracking problems. During nominal tracking mode, the trajectory tracking controller ensures asymptotic convergence to each $\xi_{j}$. Obstacle detection will trigger a conditional planning mode governed by the distance assessment. Let $d_{\mathrm{curr}}$ quantify the arc-length along current sub-path $\xi_{j}$, and $\tau_{\mathrm{path}}$ denote the switching threshold. As shown in Figure 7, the viable target region Γ follows a threshold-based switching criterion:

(14) $$\Gamma =\left\{\begin{array}{ccc} \left\{\left.p\right|p\in \xi_{j+1}\right\} & , & d_{\mathrm{curr}}\geq \tau_{\mathrm{path}}\\ \left\{\left.p\right|p\in \xi_{j}\wedge ∥P_{\mathrm{curr}}-p∥>∥P_{\mathrm{curr}}-P_{\mathrm{ob}}∥\right\} & , & d_{\mathrm{curr}}<\tau_{\mathrm{path}} \end{array}\right.$$

where $P_{\mathrm{curr}}\in R^{2}$ and $P_{\mathrm{ob}}\in R^{2}$ represent the robot/obstacle position, with ||·|| denoting the Euclidean metric.

#### 3.1.3. Design of the Sub-Goal Evaluation Function

At each planning iteration, we define the candidate sub-target selection as a evaluation problem over the feasible set Γ. The candidate point with the smallest cost in the region Γ will be selected as the local sub-target point at the current moment. The composite cost functional cost(·) integrates three critical navigation metrics:

(15) $$\text{ob-cost}\left(p_{i,\;\Gamma }\right)=\left\{\begin{array}{ccc} ob_{\max} & , & d_{\mathrm{ob}}\left(p_{i,\;\Gamma }\right)\leq \tau_{\mathrm{ob}}\\ \frac{1}{d_{\mathrm{ob}}\left(p_{i,\;\Gamma }\right)} & , & d_{\mathrm{ob}}\left(p_{i,\;\Gamma }\right)>\tau_{\mathrm{ob}} \end{array}\right.$$

(16) $$\text{dist-cost}\left(p_{i,\;\Gamma }\right)=∥P_{\mathrm{curr}}-p_{i,\;\Gamma }∥$$

(17) $$\text{yaw-cost}\left(p_{i,\;\Gamma }\right)=1-\cos\left(\Delta \varphi \left(p_{i,\;\Gamma }\right)\right)$$

**Figure 8:**
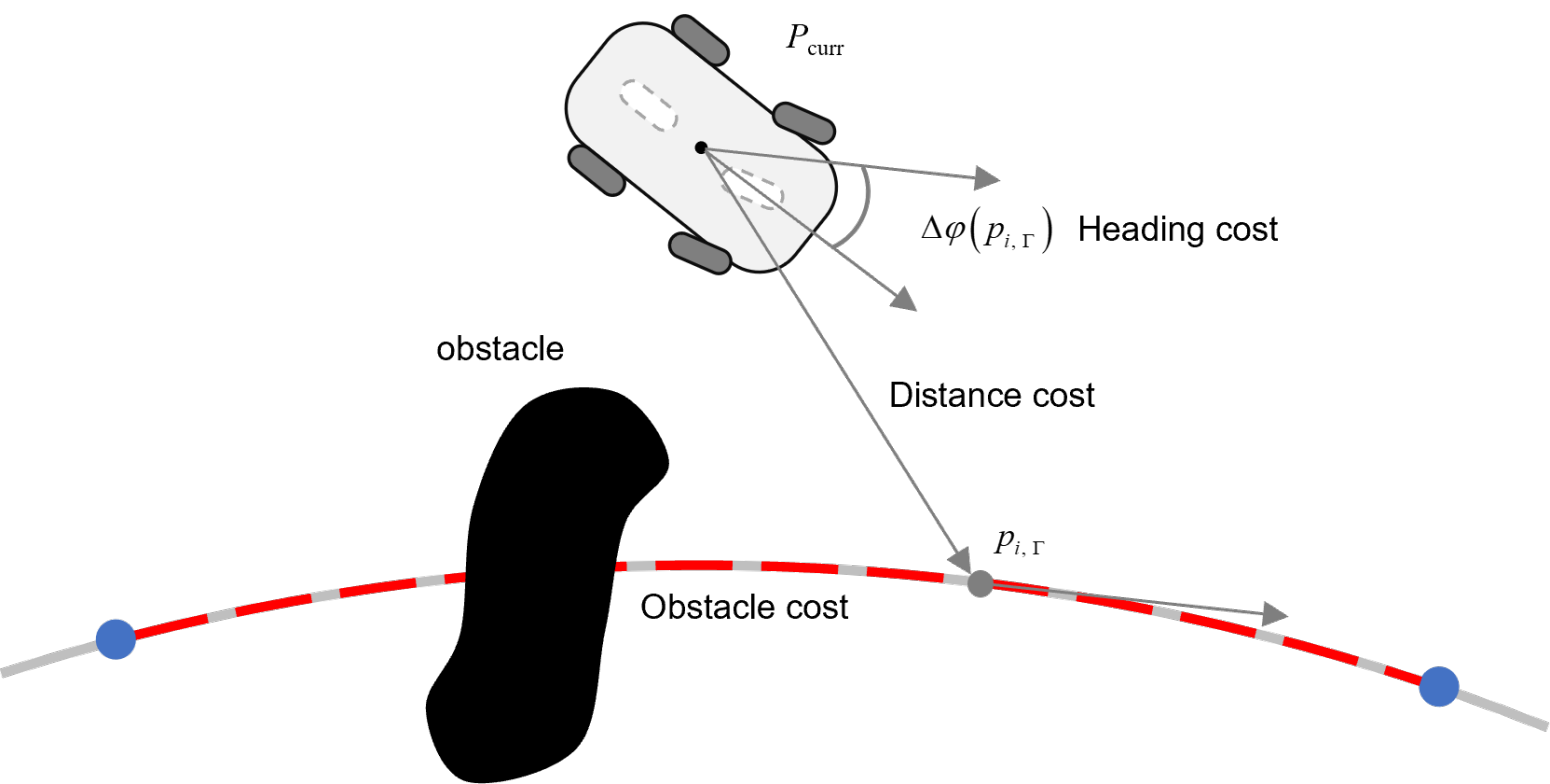
Local target point selection strategy.

As shown in Equation (18) and Figure 8, the function used to evaluate each target point $p_{i,\;\Gamma }$ can be expressed as:

(18) $$\mathrm{cost}\left(p_{i,\;\Gamma }\right)=w_{b}\cdot \text{ob-cost}\left(p_{i,\;\Gamma }\right)+w_{t}\cdot \text{dist-cost}\left(p_{i,\;\Gamma }\right)+w_{y}\cdot \text{yaw-cost}\left(p_{i,\;\Gamma }\right)$$

where $w_{b}$, $w_{t}$ and $w_{y}$ are the weight coefficients of different sub-evaluation function. $d_{\mathrm{ob}}\left(p_{i,\;\Gamma }\right)$ denotes the distance of the to-be-selected target from the obstacle, $\tau_{\mathrm{ob}}$ and $ob_{\max}$ are the predefined thresholds. $\Delta \varphi \left(p_{i,\;\Gamma }\right)$ is the heading angle difference between current position and the target point.

**Figure 9:**
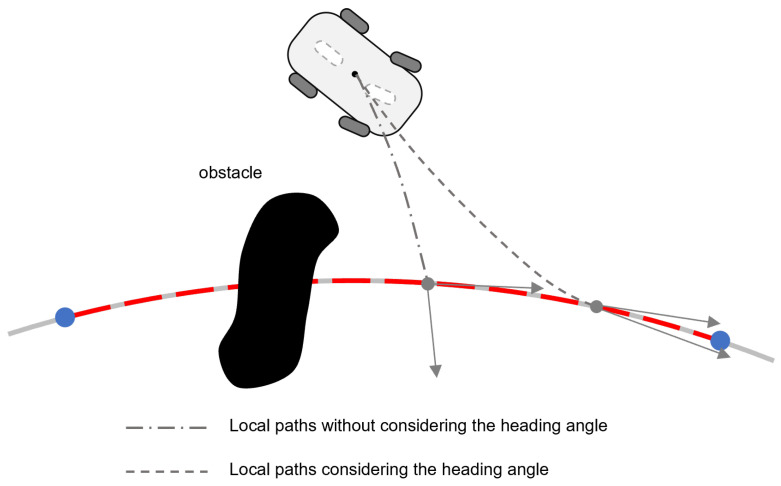
The role of the heading cost function.

**Remark 2.**  *The formulation presented in Equation (15) demonstrates that proximal positioning of sub-target points relative to obstacles induces elevated cost values, thereby preventing path planning failures caused by obstacle occlusion of the target location. This cost mechanism simultaneously eliminates sharp turns associated with the proximity of local sub-target points to obstacles, improving motion smoothness. Furthermore, to guarantee fast reintegration of the mobile robot into the normal navigation mode after obstacle avoidance, spatial proximity between the local sub-target and the current position must be constrained, as presented in Equation (16). The heading cost (Equation (17)) primarily addresses transitional continuity between local path segments and the global path. Without this orientational component, the path planner will exhibit preferential selection of geometrically proximal targets relative to the initial planning position. Under such conditions, local paths asymptotically approach orthogonality relative to the global path’s directional vector at the target node, as illustrated in Figure 9, which significantly compromises motion stability during transitional maneuvers.*

### 3.2. Trajectory Tracking Controller with MPC

#### 3.2.1. Design of Objective Function and Constraints

MPC derives optimal control inputs through real-time optimization, where the objective function and constraint formulation critically determine controller performance. The design prioritizes tracking accuracy for reference trajectories while simultaneously ensuring control continuity through bounded actuation variations to mitigate system oscillations. This dual-focused approach enables precise and stable trajectory tracking, mathematically expressed as:

(19) $$\begin{array}{cc} \; & J={∥\tilde{Y}∥}_{Q}^{2}+∥\Delta \tilde{U}{∥}_{R}^{2}\\ \; & =\sum_{i=1}^{N_{p}}{∥\eta \left(k+i|k\right)-\eta_{r}\left(k+i|k\right)∥}_{Q}^{2}+\sum_{i=0}^{N_{c}-1}{∥u\left(k+i|k\right)-u_{r}\left(k+i|k\right)∥}_{R}^{2}\\ \; & =\sum_{i=1}^{N_{p}}{\left(\eta \left(k+i|k\right)-\eta_{r}\left(k+i|k\right)\right)}^{T}Q\left(\eta \left(k+i|k\right)-\eta_{r}\left(k+i|k\right)\right)\\ \; & +\sum_{i=0}^{N_{c}-1}{\left(u\left(k+i|k\right)-u_{r}\left(k+i|k\right)\right)}^{T}R\left(u\left(k+i|k\right)-u_{r}\left(k+i|k\right)\right) \end{array}$$

where *Q*, *R* represent the positive-definite weight matrices, and $N_{p}$ and $N_{c}$ represent the prediction and control horizon. $\eta_{r}={\left[x_{r},y_{r},\theta_{r}\right]}^{T}$ and $u_{r}={\left[v_{r},\omega_{r}\right]}^{T}$ denotes the reference state and control input, respectively.

The objective function incorporates control variation penalties, imposing progressive cost escalation with increasing actuation changes to discourage abrupt adjustments. Nevertheless, the mobile robot’s mechanical limitations necessitate strict bounds on both the control input and its rate:

(20) $$u_{\min}\leq u\leq u_{\max}$$

(21) $$\Delta u_{\min}\leq \Delta u\leq \Delta u_{\max}$$

where $u_{\min}$ and $u_{\max}$ represent the minimum and maximum executable control values dictated by the actuation system. Equation (21) constrains the control rate Δu, with $\Delta u_{\min}$ and $\Delta u_{\max}$ enforcing bounds on permissible actuation variations per sampling interval. These limitations ensure compliance with the robot’s dynamic response capabilities while preventing mechanical stress caused by abrupt control commands.

Then, the optimal control problem for path tracking can be formulated as:

(22) $$\min_{u}\;J\left(\eta ,u\right)$$

subject to:

(23a) $$\begin{array}{cc} \; & \dot{\eta }=f\left(\eta ,u\right) \end{array}$$

(23b) $$\begin{array}{cc} \; & \eta \left(0\right)=\eta_{0} \end{array}$$

(23c) $$\begin{array}{cc} \; & u\in U \end{array}$$

where Equation (23a) enforces the state constraint according to the system dynamics with f(·) defining the nonlinear state transition function and the initial state $\eta_{0}$. The feasible set *U* is defined in Equations (20) and (21).

#### 3.2.2. Curvature-Adaptive Reference Point Adjustment Mechanism

Upon obstacle detection, the locally planned path might undergo abrupt curvature variations such as sharp turns. In such scenarios, conventional controllers cannot respond promptly to these sudden changes due to their inability to anticipate upcoming road conditions. This could result in a significant tracking error from the reference path, potentially leading to unintended collisions with obstacles, even when a collision-free local path is successfully generated. To solve this problem, this section develops a curvature-adaptive reference point adjustment mechanism. When a detected curvature variation exceeds a predefined threshold, a forward-looking path point is activated as the preview-enabled reference, improving the prediction capability regarding the change in the path. During curvature stabilization phases, the reference point exponentially converges to the nearest point $p_{\mathrm{near}}$, preserving path tracking continuity.

Before further discussion, the forward-looking distance $d_{f}$, the out-of-segment distance $d_{on}$, and the on-segment distance $d_{out}$ are introduced. The forward-looking distance evaluates the mobile robot’s ability to predict the path ahead and is the basis for selecting the reference point; the out-of-segment distance $d_{on}$ refers to the distance between the closest point $p_{\mathrm{near}}$ on the global path ξ and the mobile robot at the current moment; and the up-segment distance $d_{out}$ defines the the distance from the actual reference point $p_{r}$ to the nearest reference point $p_{\mathrm{near}}$:

(24) $$d_{on}=∥P_{\mathrm{curr}}-p_{i}∥,p_{i}\in \xi$$

(25) $$d_{out}=\sum ∥p_{i+1}-p_{i}∥,p_{i}\in \xi^{\prime }$$

(26) $$\xi^{\prime }=\left\{p_{\mathrm{near}},\cdots ,p_{r}\right\}$$

(27) $$d_{f}=d_{on}+d_{out}$$

The forward-looking distance exhibits significant correlation with reference point selection in trajectory tracking systems. Paths demonstrating high curvature variance demand extended preview distances to proactively compensate for abrupt curvature transitions, thereby enhancing path tracking robustness. Conversely, low-curvature paths permit reduced preview horizons through curvature-adaptive scaling. Based on this, an iterative update strategy is defined such that the forward-looking distance varies with the path curvature:

(28) $$d_{f}\left(k+1\right)=d_{f}\left(k\right)+\mathrm{sgn}\left(\delta_{c}-\kappa_{1}\right)\times \max\left(\delta_{c},\kappa_{1}\right)$$

with

(29) $$\delta_{c}=\kappa_{2}\left|c_{r}-c_{r+1}\right|$$

(30) $$d_{\mathrm{fmin}}\leq d_{f}\left(k\right)\leq d_{\mathrm{fmax}}$$

(31) $$\mathrm{sgn}\left(y\right)=\left\{\begin{array}{ccc} 1 & , & y>0\\ 0 & , & y=0\\ -1 & , & y<0 \end{array}\right.$$

where $c_{r}$ is the curvature of the path at the current reference point, and $\delta_{c}$ is associated with the curvature variance. $\kappa_{i}\left(i=1,2\right)$, $d_{\mathrm{fmin}}$, and $d_{\mathrm{fmax}}$ are positive adjustable parameters. The sign function sgn(·) determines whether the value of the forward-looking distance $d_{f}$ increases or decreases.

**Remark 3.**  *The forward-looking distance $d_{f}\left(k+1\right)$ is dynamically determined by the current distance $d_{f}\left(k\right)$ and curvature variation $\delta_{c}$. This strategy operates based on path characteristics: (1) high-curvature-variation segments (typical in local obstacle-avoidance paths with abrupt curvature transitions, e.g., sharp turns); and (2) low-curvature-variation segments (common in global reference paths composed of simple geometric curves). For local path tracking, $d_{f}$ progressively adjusts to $d_{fmax}$ through monotonic increments, enhancing path prediction accuracy to mitigate tracking errors and collision risks. Conversely, in global path tracking, $d_{f}$ asymptotically converges to $d_{fmin}$ via controlled reduction, optimizing computational efficiency, which will be further discussed at the end of this section.*

This adaptive adjustment of the forward-looking distance is achieved by comparing the curvature variation $\delta_{c}$ with a predefined threshold $\kappa_{1}$. The max(·) function determines the larger value between these two parameters, while the sgn(·) function regulates the direction of distance adjustment: when $\delta_{c}<\kappa_{1}$, in smooth global paths, the negative sgn(·) function triggers a gradual reduction in the look-ahead distance; conversely, when $\delta_{c}>\kappa_{1}$ in local paths with significant curvature variations, the positive sgn(·) function initiates a progressive increase. This mechanism enables self-adaptive switching between global and local path tracking. Based on this, the curvature-adaptive reference point adjustment mechanism can be expressed as:

(32) $$p_{r}=\left\{\begin{array}{ccc} \mathrm{argmin}\left(d_{out}-∥p_{\mathrm{near}}-p_{i}∥\right),\forall p_{i}\in \zeta & , & d_{f}>d_{\min}\\ p_{\mathrm{near}} & , & d_{f}=d_{\min} \end{array}\right.$$

**Figure 10:**
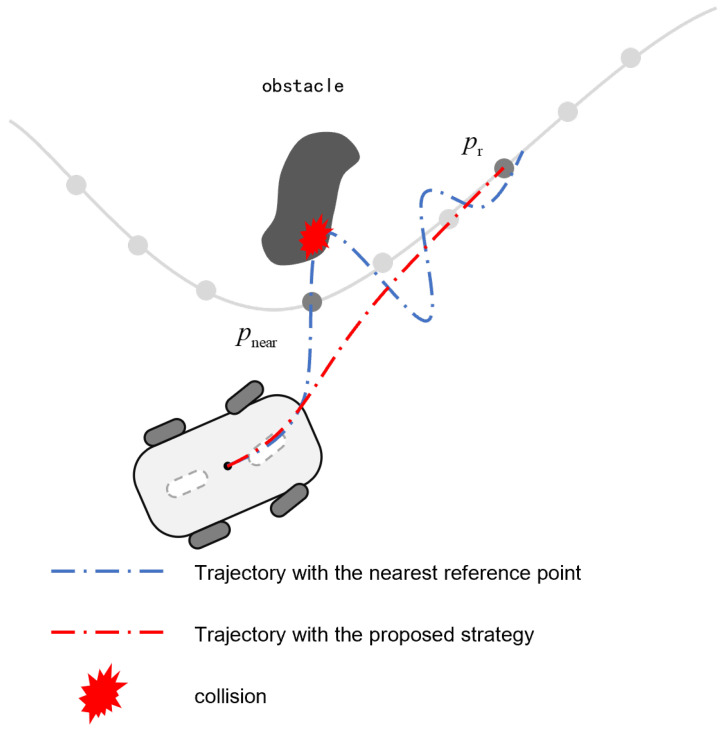
The proposed strategy reduces the possibility of accidental collision due to poor control performance.

**Remark 4.**  *This predictive curvature adaptation enables the mobile robot to anticipate path curvature variations, enhancing path tracking accuracy through improved reference path alignment. Simultaneously, it mitigates steering oscillations caused by exclusive reliance on the nearest reference point ($p_{near}$) by avoiding excessive steering maneuvers. The integrated approach thereby reduces collision risks associated with trajectory tracking inaccuracies, as shown in Figure 10.*

Adjustments in reference points may render the original prediction horizon inadequate for current operational requirements. Extended prediction horizons intensify computational complexity in optimal control problem solving, while reduced horizons impair predictive capability of upcoming paths, compromising anticipatory control input generation and tracking accuracy. Optimal performance is achieved by strategically balancing the prediction horizon to enhance tracking precision with acceptable computational cost increments, as determined by the following equation:

(33) $${\tilde{N}}_{p}=\frac{d_{f}}{v_{c}\times T}$$

(34) $$N_{p}=\min\left\{\max\left\{{\tilde{N}}_{p},N_{\mathrm{pmin}}\right\},N_{\mathrm{pmax}}\right\}$$

where $v_{c}$ denotes the current velocity of the mobile robot, *T* denotes the sampling period, and $N_{\mathrm{pmin}}$ and $N_{\mathrm{pmax}}$ are the set minimum and maximum prediction horizon, respectively.

Additionally, it is assumed in this paper that the control horizon is correlated with the prediction horizon:

(35) $$N_{c}=\mu N_{p},\mu \in \left(0,1\right)$$

Generally, the proposed framework demonstrates two novel advancements over conventional hierarchical control architectures: The standard DWA is enhanced through evaluation function refinement and sub-target selection optimization to guarantee trajectory feasibility while maintaining control system compatibility; simultaneously, curvature-aware adaptation dynamically adjusts reference points based on local path geometry, significantly enhancing control precision.

## 4. Experiment

**Figure 11:**
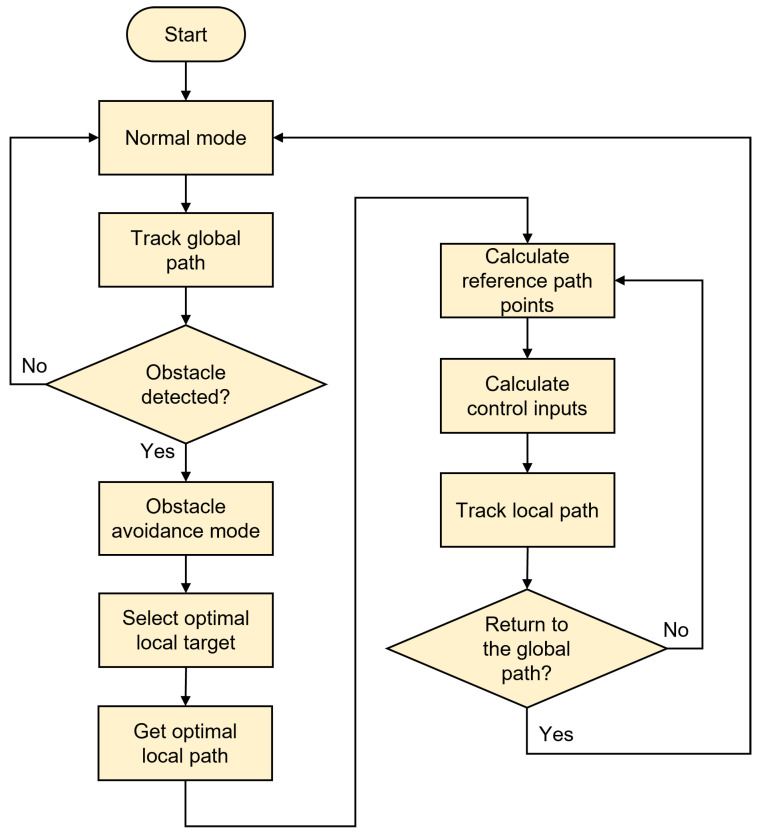
The overall flow of the proposed method.

This section evaluates the proposed hierarchical planning-control framework through dual-aspect comparative experiments, rigorously assessing path planning efficacy and trajectory tracking precision to validate the performance advantages of the proposed methodology. The general flow of the proposed method is shown in Algorithm 1 and Figure 11, and the algorithmic parameters are listed in Table 1.
**Algorithm 1** DWA-MPC Hierarchical Navigation Framework**Require:** Global path *G*, Robot state *X***Ensure:** Control command u1:**loop**2:    Local map *M*, Lidar_cloud←SENSORUPDATE()3:    Collision_flag←EVALUATECOLLISIONRISK(Lidar_cloud,X)4:    **if** Collision_flag **then**               ▹ Local Replanning via DWA5:        Candidate_goals←GENERATESUBGOALS(G,X,M,radius=2.0m)6:        Optimal_subgoal←argmaxSCORESUBGOALS(Candidate_goals,X,M)7:        τ←DWAPLANNER(X,Optimal_subgoal,M)8:        MPC.ReferenceTrajectory←τ9:        $R\mathrm{ESET}\mathrm{MPCH}\mathrm{ORIZONS}(N_{p},N_{c})$10:    **else**11:        MPC.ReferenceTrajectory←G12:    **end if**                           ▹ Adaptive MPC Tracking13:    **if** MPC_execution_cycle **then**14:        Current_ref_point←FINDNEARESTPOINT(MPC.ReferenceTrajectory,X)15:        κ←COMPUTEOPTIMALREFERENCE(MPC.ReferenceTrajectory,Current_ref_point)16:        $\left(N_{P_{\mathrm{adapt}}},N_{C_{\mathrm{adapt}}}\right)\leftarrow A\mathrm{DJUST}H\mathrm{ORIZONS}\left(\kappa \right)$17:        $u_{\mathrm{opt}}\leftarrow S\mathrm{OLVE}MPC\left(X,\kappa \right)$18:        $A\mathrm{PPLY}C\mathrm{ONTROL}(u_{\mathrm{opt}}\left[0\right])$19:    **end if**20:**end loop**

### 4.1. Local Path Planning Layer

**Figure 12:**
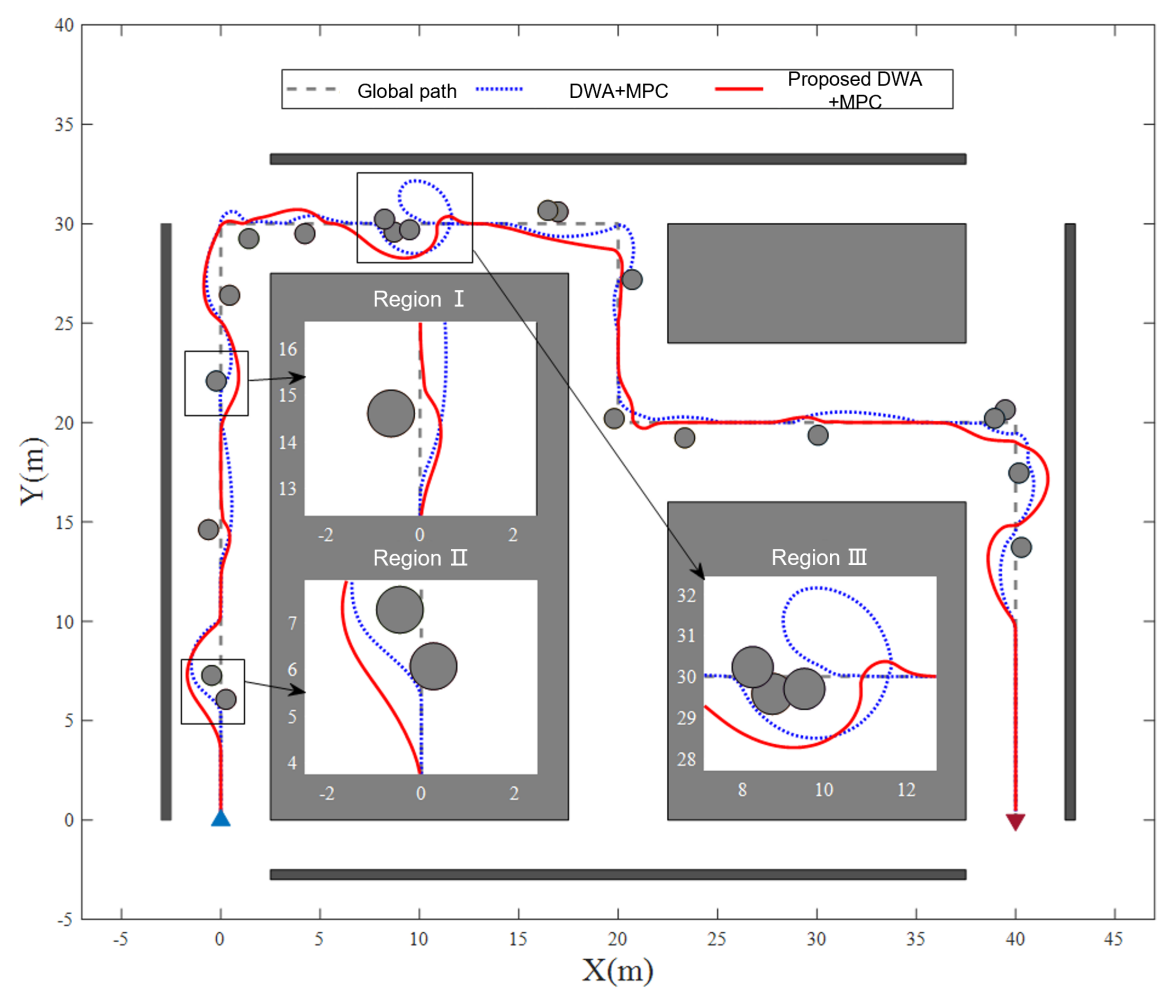
Validation results of the proposed local path planning layer.

To validate the proposed DWA, random obstacles are generated along the global path as shown in Figure 12, the gray circles represent static obstacles in the robot’s navigation environment. The radius of the gray circles is determined by the obstacle radius (0.2 m), the robot radius (0.15 m), and a predefined safety margin of 0.1 m. These obstacles simulated common indoor navigation challenges (e.g., pillars, furniture). The improved method is benchmarked against the standard DWA, with both methods coupled to the proposed MPC trajectory tracker. Statistical performance metrics—including mean path length and planning success rate—are derived from multiple experimental trials to quantify algorithm efficacy.

Figure 12 demonstrates the local path trajectories generated by both standard and enhanced DWA under identical environmental conditions. The proposed method achieves superior path smoothness by integrating a curvature continuity term in its cost function to explicitly account for geometric consistency during local path generation. This is evidenced by the robot’s gradual transition maneuvers around obstacles compared to abrupt directional changes observed in the baseline method. Region II in Figure 12 shows the mobile robot’s seamless transition from global to local path navigation, replacing abrupt steering maneuvers near obstacles with curvature-consistent path following. This anticipatory behavior mitigates collision risks induced by path curvature discontinuities during trajectory tracking control.

Regions I and III demonstrate the practical effects of the adaptive sub-target selection strategy. The strategy significantly outperforms the standard fixed-length strategy by simultaneously optimizing distance and heading angle continuity between local targets and the robot’s current position. As evidenced in Region I, the proposed approach enables faster global path recovery after obstacle avoidance. This mitigates tracking complexity through smooth local–global path transitions. Conversely, the standard DWA induces persistent global path divergence, and the robot takes a longer time to return to the global path. The obstacle-proximity awareness in local sub-target selection prevents goal occupancy conflicts, as demonstrated in Region III, by enforcing minimum clearance thresholds between targets and obstacles. Standard DWA often triggers circular search patterns near obstructed targets due to persistent infeasible path generation, invariably resulting in planning failures. Conversely, the proposed method proactively selects collision-free targets through obstacle distance optimization, thereby resolving the inherent conflict between obstacle avoidance and goal convergence to guarantee planning feasibility.

As quantified in Table 2, through 20 randomized obstacle environments, the proposed method achieves a 8.58 m reduction in average path length compared to the traditional DWA, significantly enhancing task completion efficiency. Concurrently, the planning success rate improves from 60% (12/20) to 95% (19/20), demonstrating substantial performance gains in complex navigation scenarios.

### 4.2. Path Tracking Layer

In this section, the proposed DWA will be selected as the basic local planning method, and a series of comparative experiments will be carried out using the standard MPC with $N_{p}=10$, a PID controller (P = 1, I = 0.001, D = 20), and the proposed MPC as the tracking control methods. The actual trajectory output results are jointly determined by both the trajectory tracking control layer and the local path planning layer.

**Figure 13:**
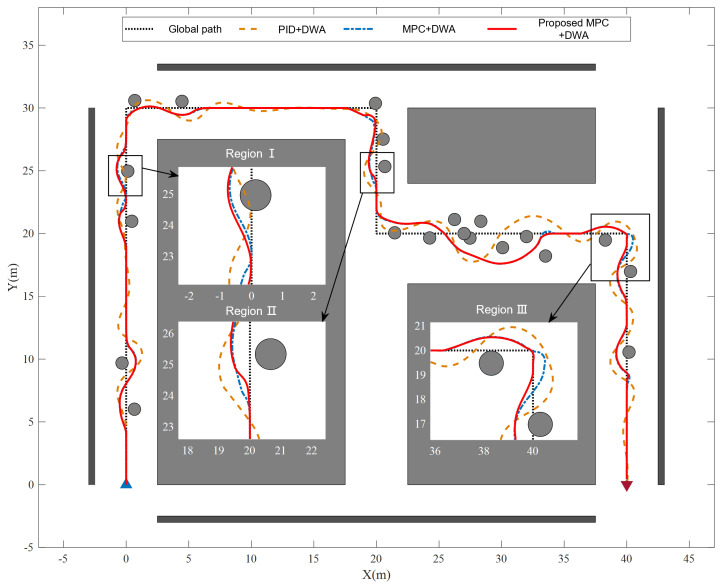
Validation results of the proposed path tracking layer.

**Figure 14:**
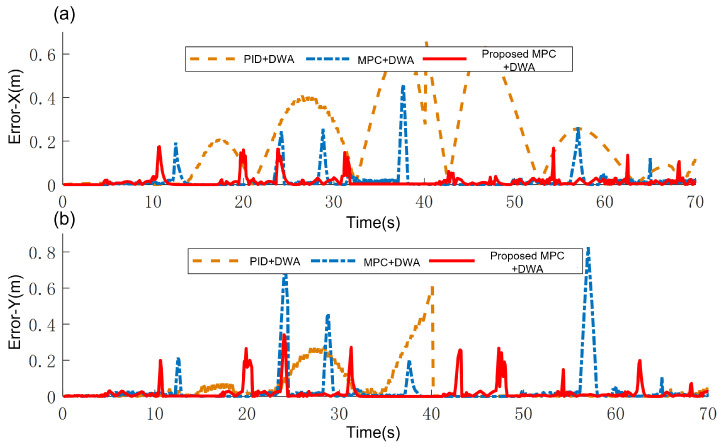
Tracking error. (**a**) Error in X direction. (**b**) Error in Y direction.

Figure 13 gives the output trajectories of the mobile robot under different tracking methods. Despite successful collision-free path planning, the PID controller exhibits significant trajectory oscillations during execution, ultimately causing collisions due to unstable tracking performance—as demonstrated by the failed maneuver in Region I of Figure 13. The curvature-adaptive MPC outperforms both the standard MPC and PID controllers in high-curvature scenarios (e.g., obstacle-avoidance paths in Region II and sharp turns in Region III), with smoother trajectory and smaller tracking errors (Figure 13). The tracking errors is shown in Figure 14.

The Integral Absolute Error (IAE) metric is adopted to evaluate the tracking accuracy:

(36) $$\mathrm{IAE}=\int_{0}^{t_{\mathrm{total}}}\left|error(t)\right|\;dt$$

where error(t) represents instantaneous tracking error. As per Table 3, the proposed MPC method reduces x-direction errors by 56.04% compared to PID control and 44.91% versus standard MPC, with corresponding y-direction error reductions of 49.10% and 44.26%, respectively. These results confirm the framework’s superior tracking precision.

**Figure 15:**
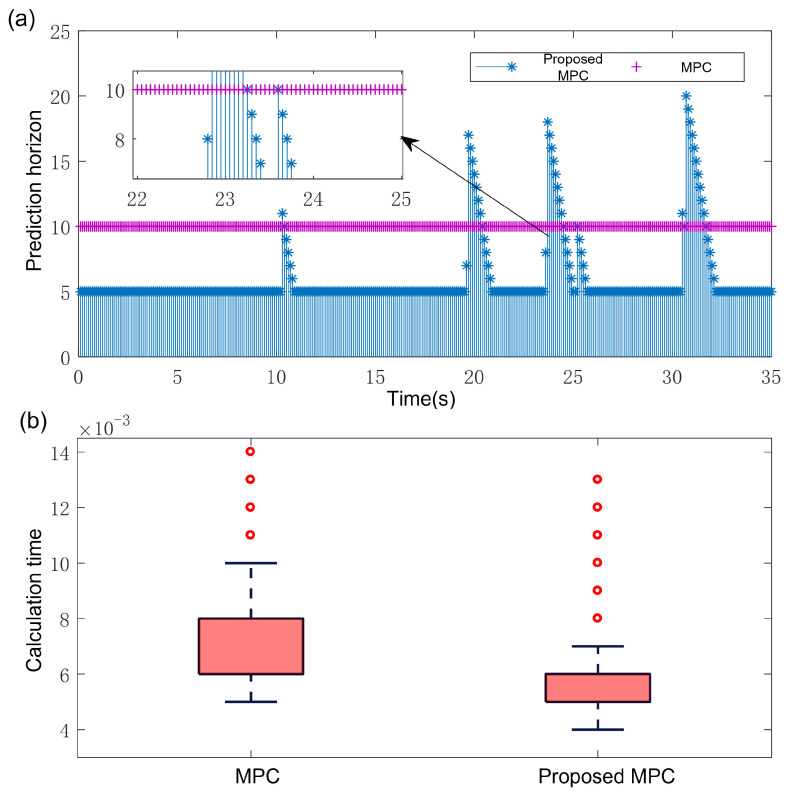
Results. (**a**) Variation in the prediction horizon of different methods. (**b**) Comparison of the calculation time.

Figure 15a illustrates the dynamic evolution of prediction horizons for both the standard and proposed methods. Prediction horizon expansion correlates with sharp curvature transitions in reference paths. This adaptation enhances tracking precision during critical avoidance maneuvers while enabling rapid convergence to minimal permissible horizons upon normal mode recovery, thereby balancing computational efficiency. Figure 15b reveals the calculation time distribution. Due to the adaptive horizon changing strategy, the average prediction horizon is shorter, thus the computational burden is smaller. The proposed method achieved a 36.6% reduction in maximum computation time and 23.5% reduction in average computation time. Overall, the proposed MPC reduces the computational burden while ensuring high tracking accuracy.

### 4.3. Real-World Experiments

**Figure 16:**
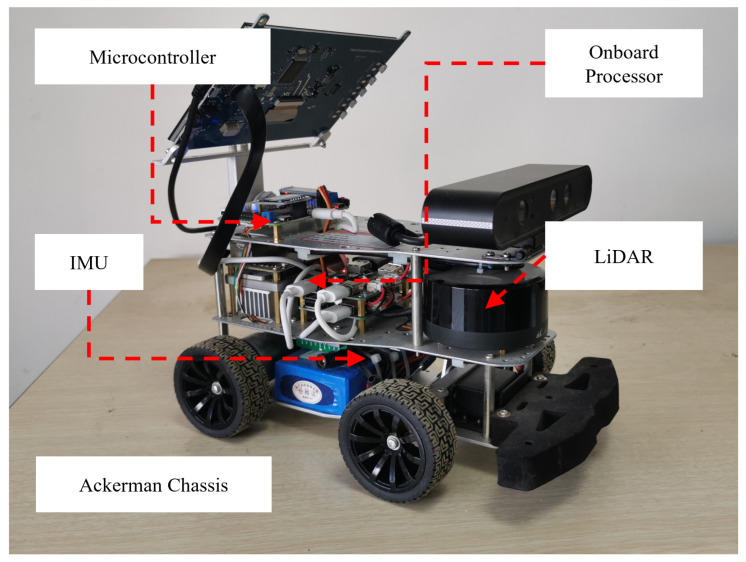
Experimental Platform.

**Figure 17:**
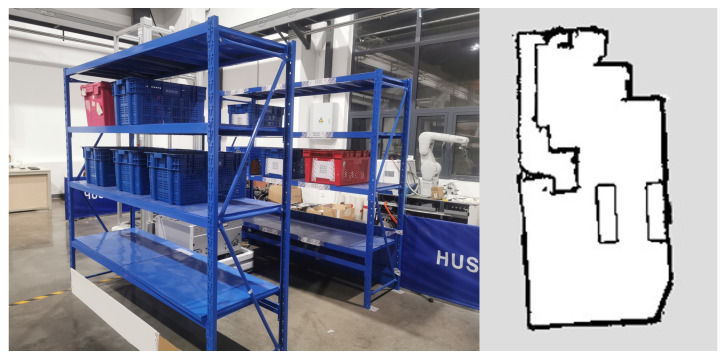
Experimental scenario.

In this section, the proposed method will be validated using the experimental platform (shown in Figure 16), while the warehouse (shown in Figure 17) is selected as the experimental scenario. The experimental platform comprises an Ackermann-steering robotic system specifically designed for autonomous navigation. This physical prototype integrates an NVIDIA Jetson TX1 embedded computing unit with a Robot Operating System (ROS)-based architecture. The optimization problem is formulated and solved via the CasADi framework [33] with a nonlinear programming solver, maintaining a 10 Hz control loop frequency. For environmental perception, a planar LiDAR sensor with a 5 m maximum ranging capability and an inertial measurement unit (IMU) provide obstacle localization and ego-state estimation, respectively, with raw point cloud data processing implemented through Point Cloud Library. All software components were developed using the C++ programming language with ROS Melodic integration, ensuring real-time performance optimizations.

#### 4.3.1. Scene I

**Figure 18:**
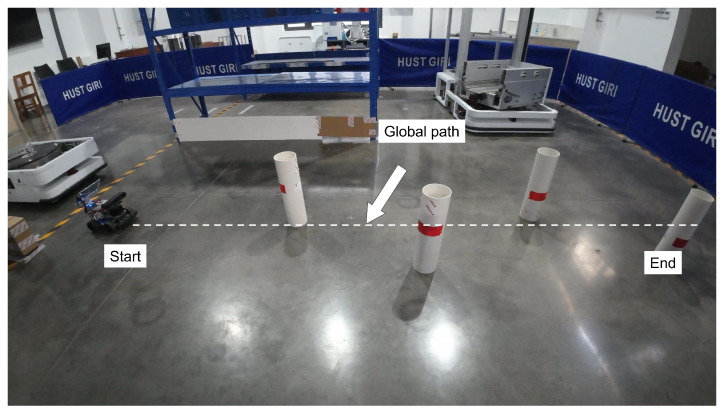
Scene I.

**Figure 19:**
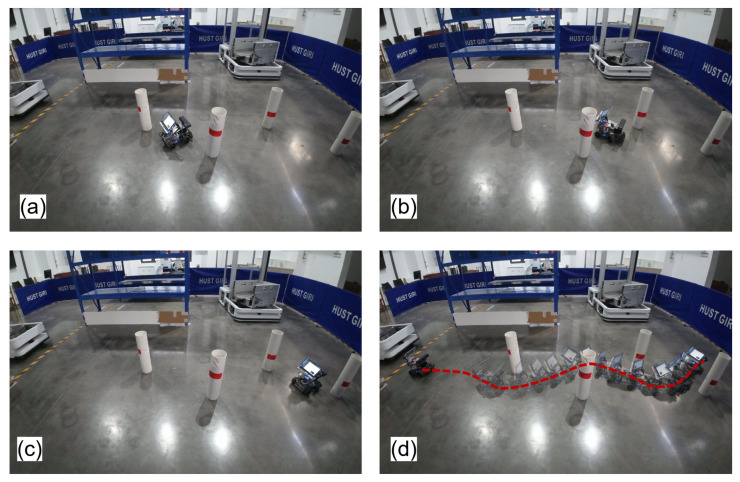
Experimental results in scene I. (**a**) Narrow passage navigation. (**b**) Robot moves between two obstacles. (**c**) Robot bypasses the last obstacle and about to reach the end point. (**d**) The whole navigation progress. The red dashed line shows the actual trajectory of the robot.

Figure 18 shows the experimental scene in this section and Figure 19 demonstrates the hierarchical planning–control framework’s real-time obstacle response capability. Upon detecting obstacles, the system activates the local path planning layer to generate a collision-free local trajectory while executing curvature-adaptive tracking maneuvers via the proposed MPC. The whole navigation progress is shown in Figure 19d. Through the local sub-target selection strategy, the mobile robot prioritizes navigation between two obstacles (narrow passage navigation, as shown in Figure 19a,b). Compared to moving from the same side of two obstacles, this preference makes the local path closer to the global path as much as possible while ensuring safety. Figure 19c shows the mobile robot bypassing the last obstacle and about to reach the end point.

#### 4.3.2. Scene II

**Figure 20:**
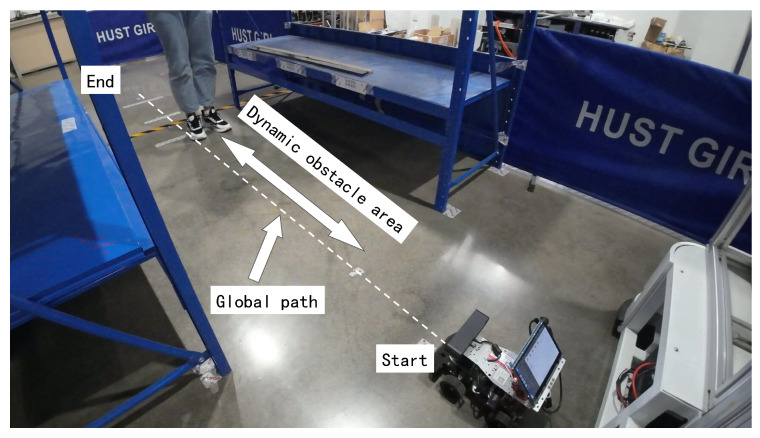
Scene II.

In scene II (Figure 20), the robot has to move along the global path from the start point to the end point, avoiding a human moving back and forth.

**Figure 21:**
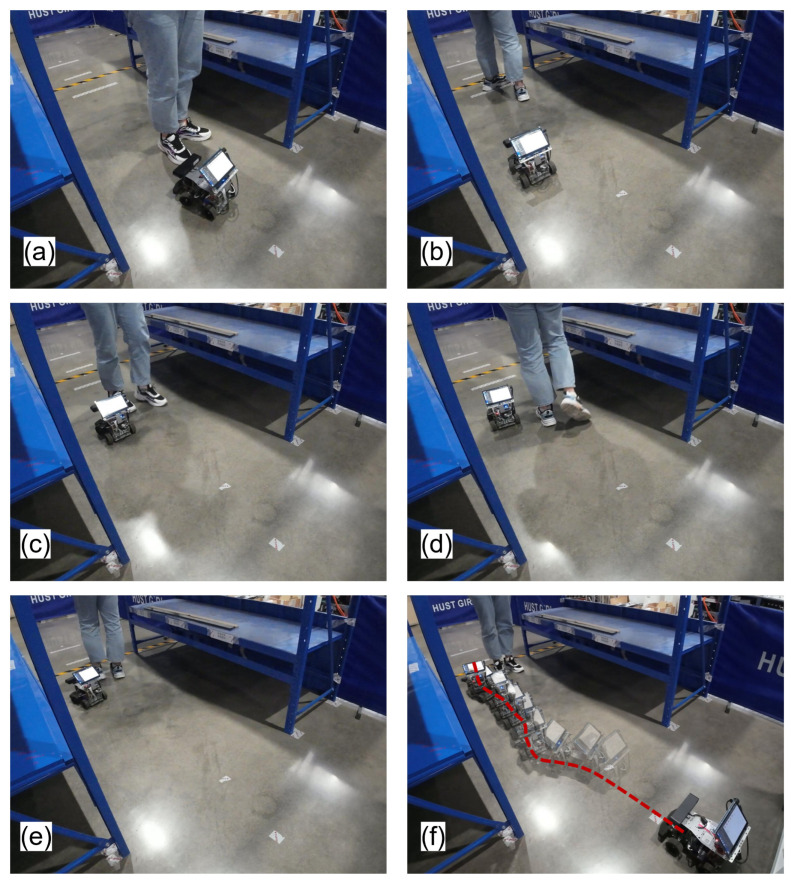
Experimental results in scene II. (**a**) Obstacle moves towards the robot. (**b**) Robot returns to global path after avoiding the obstacle. (**c**) Obstacle moves towards the robot. (**d**) Obstacle moves in the same direction as the robot. (**e**) Obstacle moves in the same direction as the robot. (**f**) The whole navigation progress. The red dashed line shows the actual trajectory of the robot.

Figure 21 illustrates the robot’s dynamic obstacle avoidance and path recovery behaviors under moving obstacle conditions. Figure 21a,c demonstrate evasive steering maneuvers triggered by approaching the obstacle, while Figure 21d,e show the avoidance behavior when the robot is moving in the same direction as the obstacle. Recovery motion to global reference trajectory after avoiding the obstacle is documented in Figure 21b. The steering logic is as follows: left-turn avoidance dominates after detecting the obstacle (Figure 21a,c,e); and right-turn recovery activates upon threat clearance (Figure 21b,d) to minimize global path deviation. The whole navigation progress is shown in Figure 21f.

**Figure 22:**
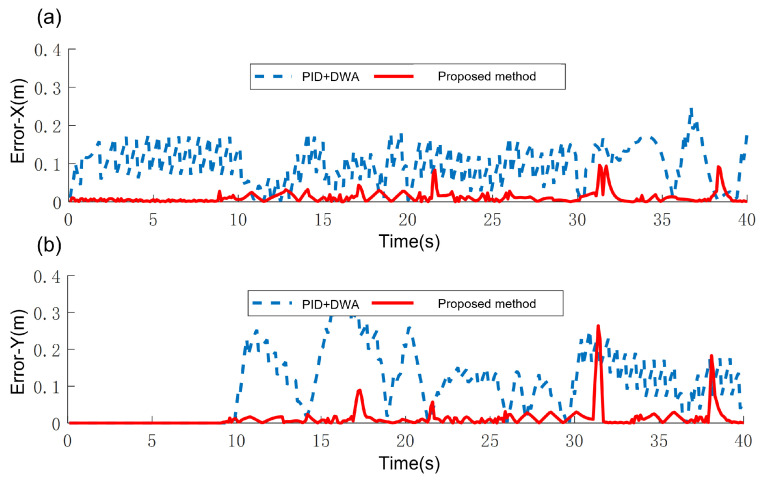
Tracking error. (**a**) Error in X direction. (**b**) Error in Y direction.

The proposed hierarchical strategy is benchmarked against PID control and baseline DWA, and the tracking error is shown in Figure 22. Quantitative analysis reveals 90.26% and 84.40% reductions in mean tracking errors (x: 0.113 m → 0.011 m; y: 0.109 m → 0.017 m), significant improvements validating the framework’s superior path-following accuracy. These precision gains directly translate to enhanced obstacle avoidance reliability, particularly critical in the space-constrained operational settings.

## 5. Conclusions

This study addresses the path planning and control challenges for mobile robots by proposing a hierarchical framework integrating local path planning and trajectory tracking control. The standard DWA is enhanced through modified evaluation functions and an adaptive sub-target selection strategy, improving planning success rates, path smoothness, and reducing tracking control complexity. An MPC-based trajectory tracking layer is developed with a curvature-adaptive reference point adjustment mechanism to enhance control accuracy during abrupt curvature variations. The prediction horizon is adaptively adjusted to balance prediction reliability and real-time computational efficiency.

Comparative experiments across diverse scenarios validate the proposed framework’s performance, demonstrating superior tracking accuracy and obstacle-avoidance performance over current approaches, establishing a new benchmark for autonomous mobile systems requiring both efficiency and safety guarantees. Specifically, it achieves a reduction in average path length alongside dramatically improved planning reliability, substantially enhancing operational efficiency. The customized MPC formulation outperforms both PID control and standard MPC in tracking precision, reducing lateral errors, with absolute tracking accuracy reaching millimeter-level precision. These advancements are achieved while concurrently reducing computational load, enabling real-time performance crucial for dynamic environments.

Future research will prioritize two extensions: (1) real-time planning-control co-optimization under dynamic uncertainties via probabilistic obstacle intention modeling, enhancing adaptability to unpredictable dynamic obstacle motions and sensor noise; and (2) distributed multi-agent collaborative navigation integrating priority-aware coordination protocols and formation control to address large-scale logistics scenarios. These advancements will be validated in human–robot shared workspaces and industrial digital twins, and comparative experiments with state-of-the-art dual-layer navigation frameworks will be conducted, bridging theoretical innovation with real-world deployment challenges.

## Figures and Tables

**Table 1:** Parameters.

Parameter	Symbol	Value	Parameter	Symbol	Value
DWA_Distance weight	$w_{d}$	0.2	SubGoal_Obstacle weight	$w_{b}$	0.5
DWA_Velocity weight	$w_{v}$	0.1	SubGoal_Distance weight	$w_{t}$	0.3
DWA_Obstacle weight	$w_{o}$	0.4	SubGoal_Heading weight	$w_{y}$	0.2
DWA_Curvature weight	$w_{c}$	0.3	Max Prediction horizon	$N_{pmax}$	20
Max velocity (linear)	$v_{max}$	0.3 m/s	Min Prediction horizon	$N_{pmin}$	5
Max velocity (angular)	$w_{max}$	1 rad/s	MPC_weight	Q	0.8
Horizon parameter	μ	0.4	MPC_weight	R	0.2

**Table 2:** Average length and success rate of different methods.

	Traditional DWA	Proposed DWA
Average path length (m)	111.9768	103.3942
Success rate (%)	60	95

**Table 3:** Quantitative analysis.

	IAE-x	IAE-y
PID + DWA	25.5889	30.0039
MPC + DWA	20.4195	27.4022
Proposed MPC + DWA	11.2496	15.2733

## Data Availability

Data are contained within the article.
